# Moving from capstones toward cornerstones: successes and challenges in applying systems biology to identify mechanisms of autism spectrum disorders

**DOI:** 10.3389/fgene.2015.00301

**Published:** 2015-10-07

**Authors:** Nathan Kopp, Sharlee Climer, Joseph D. Dougherty

**Affiliations:** ^1^Department of Genetics, School of Medicine, Washington University in St. Louis, St. LouisMO, USA; ^2^Department of Psychiatry, School of Medicine, Washington University in St. Louis, St. LouisMO, USA; ^3^Department of Computer Science and Engineering, Washington University in St. Louis, St. LouisMO, USA

**Keywords:** autism, ASD, WGCNA, systems biology, network analysis, review, CSEA

## Abstract

The substantial progress in the last few years toward uncovering genetic causes and risk factors for autism spectrum disorders (ASDs) has opened new experimental avenues for identifying the underlying neurobiological mechanism of the condition. The bounty of genetic findings has led to a variety of data-driven exploratory analyses aimed at deriving new insights about the shared features of these genes. These approaches leverage data from a variety of different sources such as co-expression in transcriptomic studies, protein–protein interaction networks, gene ontologies (GOs) annotations, or multi-level combinations of all of these. Here, we review the recurrent themes emerging from these analyses and highlight some of the challenges going forward. Themes include findings that ASD associated genes discovered by a variety of methods have been shown to contain disproportionate amounts of neurite outgrowth/cytoskeletal, synaptic, and more recently Wnt-related and chromatin modifying genes. Expression studies have highlighted a disproportionate expression of ASD gene sets during mid fetal cortical development, particularly for rare variants, with multiple analyses highlighting the striatum and cortical projection and interneurons as well. While these explorations have highlighted potentially interesting relationships among these ASD-related genes, there are challenges in how to best transition these insights into empirically testable hypotheses. Nonetheless, defining shared molecular or cellular pathology downstream of the diverse genes associated with ASDs could provide the cornerstones needed to build toward broadly applicable therapeutic approaches.

## Introduction

Autism spectrum disorder (ASD) is a pervasive developmental disorder, affecting around one of every 100 children. ASD is characterized by profound deficits in communication and social interaction as well as restricted interests and resistance to change. ASD clearly has a strong genetic component, with a 60–90% concordance between monozygotic twins. However, the disorder shows remarkable heterogeneity in the genetic risk factors. Common variant analyses have identified few reproducible associations across studies, and meta-analyses suggest that what common variants do exist likely have small individual effects (odds ratios less than 1.2) and act in a highly polygenic manner (Anney et al., 2012; Klei et al., 2012; Gaugler et al., 2014). Thus, the recent focus has been on rare variants, including copy number variations (CNVs), and exome sequence analyses (Pinto et al., 2010; Sanders et al., 2011, 2012; Chahrour et al., 2012; Malhotra and Sebat, 2012; Neale et al., 2012; O’Roak et al., 2012b; Yu et al., 2013; De Rubeis et al., 2014; Iossifov et al., 2014). These studies collectively have identified a clear role for rare and private deleterious coding mutations, both *de novo* and inherited. However, though of larger effect size, the rarity of these individual events limits statistical power. For example, while *de novo* loss-of-function mutations may collectively account for around 10% of ASD cases, any given gene might be seen to be mutated only in 2 or 3 cases out of the thousands now sequenced (Sanders et al., 2011; De Rubeis et al., 2014). Nonetheless, since 2012 a number of *de novo*, apparent loss-of-function mutations have been described that are found primarily in individuals with ASD, and a growing number of the same genes have been mutated frequently enough to indicate clear association. Ongoing efforts are poised to discover many more. Current estimates indicate there will be several hundred genes implicated by this approach when sufficient sample size is obtained (Krumm et al., 2014), in addition to the >100 genetic syndromes which already show some shared genetics or comorbidity with ASD (Betancur, 2011; Yu et al., 2013). With the number of new ASD variants being discovered the research bottleneck now is the identification of the neurobiological mechanisms by which they act. Since the genetic heterogeneity is so substantial, it is hoped that the identification of common neurobiological mechanism(s) across these diverse genetic causes may suggest some common routes to treatments.

The relatively recent advent of computational science has produced tools that enable opportunities to unveil truths that are not reachable using only theoretical or experimental approaches alone (Reed et al., 2005). Consequently, many recent scientific advancements have materialized thanks to two alternating and complementary modes of reasoning (Kell and Oliver, 2004). Discovery-driven approaches focus on *inductive* reasoning; they examine wide sources of data and attempt to define hypotheses from the emergent patterns that describe cause and effect relationships. In contrast, hypothesis-driven approaches leverage *deductive* reasoning to identify the logical consequences of a specific theory or hypothesis; consequences that can then be tested in an experimentally rigorous manner. The dawn of the genomic era, with the ability to measure the expression of thousands of genes, protein–protein interactions, epigenetic marks, etc., has produced fertile grounds for discovery-driven analyses, and many groups are leveraging these data resources in joint analyses with human genetics data for ASD to provide novel insights into any shared characteristics of the genes and potential mechanisms of this disorder. Here, we review these studies with a particular focus on what bioinformatic approaches may have indicated about the molecular or cellular mechanisms of ASD. Then, we also highlight some of the successes and the challenges facing these approaches, along with a limited number of recommendations toward possible solutions. The overall aim of this review is to spur robust, critical, and creative thinking to advance the field.

### Evolution of Discovery-Driven Applications for ASD-Related Genes

Studies of ASD genetics have evolved substantially over the last 15 years. As it was realized that common variants of large effects would be truly rare, it became evident that large sample sizes would be necessary to power both common and rare variant analyses. To amass these samples, large gene discovery projects required the coordinated efforts of hundreds of researchers with specialized expertise (clinicians, biologists, statisticians, programmers, etc.). The end results of these studies were essentially tables: tables of SNPs showing tentative association, linkage, or transmission disequilibrium (Ma et al., 2009; Wang et al., 2009; Weiss et al., 2009), or tables of CNVs (Sebat et al., 2007; Marshall et al., 2008; Bucan et al., 2009; Glessner et al., 2009; Pinto et al., 2010; Levy et al., 2011; Sanders et al., 2012), or *de novo* and recessive single nucleotide variants (SNVs; Gilman et al., 2011; Chahrour et al., 2012; O’Roak et al., 2012b; Sanders et al., 2012; Yu et al., 2013; De Rubeis et al., 2014; Iossifov et al., 2014) occurring, with some statistical confidence, in individuals with ASD and other forms of developmental delay. These tables, collectively, have provided the foundational resource to begin understanding the human biology of ASD.

The results in these tables are arguably significant enough that a study is complete when they are generated. But they are difficult to reduce to a single statement for a title, or to summarize in an abstract, and perhaps aesthetically unpleasing as a final figure. Thus, the emergence of a ‘capstone analysis.’ Early on, if only a single candidate region or two arose from a study, such an analysis might be as assessing association between a SNP and gene expression (e.g., *CDH9*) or between cases and controls for gene expression (e.g., *SEMA5A*), which were the capstone figures of two early common variant GWAS studies (Wang et al., 2009; Weiss et al., 2009). But as the tables became longer, the capstone analysis was often focused on summarizing the likely candidate genes on the table as a whole, i.e., to provide a systematic gestalt of these genes. Examples included leveraging the GOs resource to identify disproportionately represented categorical terms [e.g., Cytoskeletal elements or Rho GTPases (Pinto et al., 2010), known to regulate neurite outgrowth (Hall and Lalli, 2010)], or an attempt to organize all the resulting genes into some kind of network (**Box 1**) using other data resources. In more recent years, these capstones have expanded in scope and in effort (Gai et al., 2012; De Rubeis et al., 2014; Pinto et al., 2014), sometimes sufficiently to become companion and *post hoc* analytical manuscripts focused on finding common themes to the discovered genes, and presumably the disorder (Gilman et al., 2011; Ben-David and Shifman, 2012; Parikshak et al., 2013; Willsey et al., 2013; Krumm et al., 2014; Xu et al., 2014; Chang et al., 2015; Hormozdiari et al., 2015). In a review by Willsey, the earlier works have been characterized as initially using ‘static’ data resources to contextualize the findings, but eventually turning to more ‘dynamic’ resources such as gene expression across brain regions or cell types in the CNS (Willsey and State, 2015). As gene expression inherently includes an aspect of brain region and developmental time, they could be equally described as moving from trying to find a shared molecular pathology for these genes, to trying to find a shared regional or cellular pathology. Below, we review capstones from both types of analyses and highlight recurrent themes that may be emerging across groups.

**Box 1. Definitions of key terms.**

**Network:** A graphical representation of entities and their relationships. Entities are represented as nodes and relationships between pairs of entities, as defined by some experimental measure, are represented as weighted or unweighted edges (lines) between the corresponding node pairs. Experimental measures might include correlated expression, weighted evidence of protein–protein interaction, or number or presence of curated connections from the literature. Many of the capstone analyses used network-based tools.

**Module:** A subgraph of a network that contains nodes that are more highly interconnected to each other than to other nodes in the network. Various formal definitions exist, but we use this general intuitive definition for the purposes of this review. Modules are typically identified using clustering or graph partitioning algorithms.

**Gene set:** A group of genes that share a particular feature. Straightforward statistics exist for determining if two gene sets overlap more than expected by chance. Some examples include:

1. A candidate gene set (for example, all genes implicated in ASD in a particular study).
2. A set of genes that all belong to the same co-expression module.
3. A set of genes sharing known features as exemplified by gene ontologies categorization. For example, a set of genes sharing a molecular function (e.g., all kinases) or presumed biological process (e.g., members of the Krebb cycle metabolic pathway).
4. A set of genes expressed in a given cell type during a specific developmental period.

**Pathway:** A series of events that link molecules and leads to a final product or change in the cell or organism. There are varied uses of this word in the literature.

1. A **metabolic pathway** might be a series of enzymes that progressively alter a metabolite (for example, the Krebb cycle).
2. A **signaling pathway** is a series of molecules, usually proteins, that transmit biological information, primarily using chemical modifications to activate or inhibit signaling activity of downstream targets.
3. A **genetic pathway** is a set of genes that contribute to a common final phenotype *in a related manner*, as determined by epistatic analyses. Note that additive effects on phenotype are not sufficient to place two genes into the same pathway. To be firmly placed in the same genetic pathway, gene products must be shown to be complementary, dominant, or suppressors of one another.

For the purposes of this review, metabolic and signaling pathways are referred to and treated simply as gene sets. The term ‘pathway’ will be used to refer exclusively to genetic pathways. Note that discovery of genetic pathways historically has led to the elucidation of a corresponding specific type of pathway (e.g., a signaling pathway such as Wnt signaling), though initial definition of a genetic pathway requires no knowledge of molecular function, only measurement of an effect on a phenotype.

**Circuit:** Generally, a course along which chemical and electrical signals travel. While a cellular circuit has some analogy to a ‘molecular circuit’ or signaling pathway, here we are distinguishing between these two levels of analysis. For the purposes of this review, circuits only refer to series of interconnected neural cells that mediate a particular behavior.

#### A Shared Molecular Pathology for ASD-Related Genes?

Given a set of genes, a variety of mature tools exists for identifying disproportionately shared molecular functions for these genes, mostly based on researcher-curated collections of gene functions (e.g., GO), or empirically determined sets of protein–protein interactions, derived from literature mining or high throughput screens in simplified model systems (e.g., yeast 2-hybrid; Ashburner et al., 2000; Lage et al., 2007; Rossin et al., 2011; Szklarczyk et al., 2015). These approaches have highlighted a variety of enriched molecular functions amongst ASD related gene sets (**Table 1**). However, the utility of the results from these approaches have two limitations; they are dependent on manually curated annotations, and they do not lead directly to falsifiable hypotheses.

**Table 1:** Summary of findings regarding enriched functional categories for ASD genes.

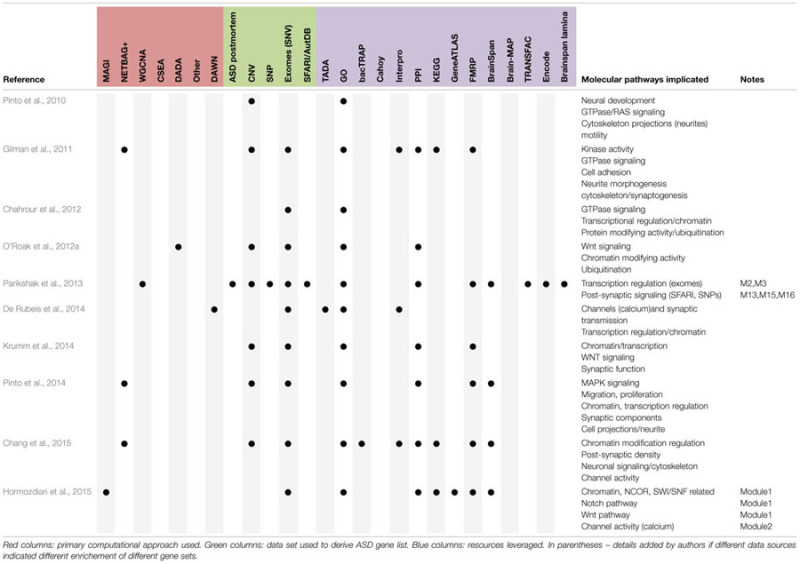

*First*, while these GO-based tools are indescribably preferable to the alternative (attempting to manually curate the literature for dozens or 100s of genes simultaneously), it is clear that they also suffer from a derivative of one of the classic barriers to unadulterated inductive reasoning described by Francis Bacon – a sort of collective version of his ‘idols of the cave.’ The term classically refers to how an individual’s interpretations of data are colored by their prior knowledge and experiences (Bacon, 1620). Likewise, GO terms are assigned based on the collective experiences of researchers, as reflected in the literature, and thus they can only be readily leveraged for well-annotated genes. In addition, even known genes may have unidentified pleiotropic molecular functions. For example, FMRP, the RNA binding protein disrupted in Fragile X syndrome, has recently been shown to also physically regulate presynaptic voltage gate potassium channels through protein–protein interactions (Deng et al., 2013), independent of any RNA binding activity. It may likewise be found that genes currently annotated as chromatin modifiers (e.g., *CHD8)* or histone deacetylase (e.g., *HDAC5*), may have as yet unknown roles in directly modifying cytoskeletal elements regulating neurite morphogenesis. Simply put: analyses based on curated knowledge cannot account for currently unknown functions.

*Second*, it is not always clear how the insights from these molecular gene set analyses might be actionable for identifying mechanistic hypotheses for ASD or developing new therapeutics. Results such as an enrichment of genes in the GO gene set 0045216 (intercellular junction assembly and maintenance), which contains 159 genes, provides limited insight on which direction to pursue. In addition, the specificity of the 159 genes in the entire GO gene set to ASD or a particular question (e.g., drug targets, causative genes, and temporal expression of ASD genes) is unknown. It has been long shown in model systems that genes that perform functions in the same genetic pathway or encode for proteins in the same protein complexes lead to similar phenotypes when disrupted, but it is not clear how closely linked a particular genetic pathway (**Box 1**) is with a given GO gene set. Thus, it would be ambitious to assume that disrupting any of the 159 genes associated with this GO gene set will lead to ASD. This is also because the 159 genes could be expressed in markedly different locations in the brain and the behavioral manifestations of such molecular disruptions will be highly dependent on the specific neural circuits that utilize each of these proteins.

In contrast, there are clear successes – that have led to purposeful experiments and meaningful treatments – arising from identifying the relevant neural circuit for a particular disorder. Note the rich variety of treatments arising from the knowledge that Parkinsonism is due to loss of dopaminergic cells of the Substantia Nigra. Long before any genes were identified that contributed to the development of this disorder, knowledge of the aﬄicted circuit (**Box 1**) led to the identification of viable treatment strategies. If the dysfunction of particular circuits in the brain manifests as explicit behavioral abnormalities (e.g., specific symptoms), then it is reasonable to assume that the shared symptomatology across distinct genetic causes of ASD implies some convergent neural circuit disruption downstream of these distinct genetic pathways. Encouragingly, if the diverse set of rare causative genetic mutations in ASD does share a common cellular or circuit mechanism, then we do not need to devise treatments for each specific rare mutation. Rather, treatments focused on correcting the common cellular dysfunction could be applied to individuals who have a variety of underlying causes, analogous to the common treatments used regardless of which genetic factor or environmental exposure was the underlying cause for a case of Parkinson’s disease. Thus, identifying common cellular circuits mediating the behavioral disruptions seen across a variety of distinct ASD genetic etiologies is essential for designing practical treatments for this disorder.

#### A Shared Cellular Pathology for ASD-Related Genes?

To address the two limitations outlined above and to attempt to identify some shared neurobiological circuit disrupted across distinct genetic causes, we and others have focused on complementary analyses leveraging gene expression data resources. As gene expression is readily measured even for unannotated genes, it is unbiased and does not suffer from the ‘idols of the cave.’ And, as gene expression varies substantially across cell types or circuits, it may be possible to implicate particular circuits by expression alone. At an extreme, a disease gene selectively expressed in a single cell type in the brain (e.g., the narcolepsy-related peptide Hypocretin found only in a population of cells in the hypothalamus; Peyron et al., 2000), clearly implicates that cell type as a vulnerable population in the disorder and any related circuits as targets for treatment. While such all-or-none expression of genes in a single cell type is rare, the logic of this ‘selective expression’ hypothesis may be somewhat extensible to a more moderate statistical enrichment of expression as well: disproportionately enriched expression of a large number of disease genes in a particular cell type or tissue could indicate a relevant anatomical intermediary of a disorder. Indeed, we have now shown that retinopathy-causing genes are disproportionately expressed in rods and cones (Xu et al., 2014). Likewise, SNPs associated with autoimmune diseases by GWAS tend to be eQTLs for genes expressed in the blood where immune cells are prevalent (Ardlie et al., 2015). And knowledge of anatomical intermediaries leads to testable hypotheses: individual cell types can be disrupted in model organisms quite readily using Cre/Lox, optogenetics, and related approaches, and behavioral consequences examined.

Before summarizing the results of the analyses leveraging expression data, it is worth noting that while gene expression data have the advantage that they are relatively unbiased for specific genes, several caveats remain. First, determination of expression levels can be affected by variations in sample collection and preparation, technician experience, equipment calibration, and choices of pre-processing algorithms, statistical tests, thresholds, microarray/RNAseq platforms, and other aspects of study design (Gudjonsson et al., 2010; Suárez-Fariñas et al., 2010). However, stringent consistency throughout the study and prudent design choices can help to ensure reasonable accuracy with regard to *relative* differences between expression levels, and these relative differences are adequate for most subsequent analyses. Second, covariates such as differences in gender, age, cause of death, time to preservation of the sample, and batch effects are sources of potential bias that are typically corrected using standard methods, such as ANCOVA (Huitema, 2005). While commonly overlooked, in order to ensure spurious relationships do not slip past these corrections, it is important that covariate information is double-checked following subsequent analyses. For example, if a co-expression module of a couple dozen genes is identified, the individuals bearing most, or all, of the expression pattern should be extracted and the degree of correlations with covariates should be determined. Third, variations in ancestry or overlooked sample relatedness can present unexpected sources of bias. An effective, albeit not always practical, way to identify either of these potential pitfalls is to collect genotype data and analyze them using packages such as Structure (Pritchard et al., 2000) and PLINK (Purcell et al., 2007). If this additional data collection is impractical, thorough screening of study participants can help alleviate these possible sources of bias. Finally, inadequate sample size can lead to serious issues as described later in this review.

If these issues are addressed, then two approaches can be taken to leverage gene expression data. The approach we took in our particular analyses were ‘top down.’ We defined sets of genes with enriched expression in different tissues based upon available body-wide RNAseq data resources (GTEX: Ardlie et al., 2015), in different cell types based on cell specific profiling technologies from mouse data (bacTRAP: Doyle et al., 2008), and profiles of human brain regions across development (Brainspan: Kang et al., 2011). We then examined the overlap of these lists with candidate disease genes, in a manner very analogous to the tools overlapping candidate gene sets with GO. However, there are weaknesses to this approach. Our use of mouse data assumes conservation of gene expression in particular cell types across mammals – a reasonable, but clearly not perfect assumption (Zeng et al., 2012). And our approach also does not explicitly leverage the correlation structure of gene expression across tissues. Likewise, human brain-region and tissue-wide data sets lose the cellular level resolution that may be most useful for identifying targets for treatments. Both data resources are limited of course to the samples that were collected, and other cell types, tissues, or perhaps key developmental windows might be absent from a particular analysis. Human data in particular have focused heavily on cortex, potentially under-representing other regions that may be of importance (e.g., hypothalamus or brainstem). Thus, these analyses are moving *toward* being potentially usable as cornerstones for developing hypotheses of the cellular mechanisms underlying ASD, and will hopefully provide additional insights as more data become available.

A complementary set of ‘bottom up’ data-driven studies address some of these concerns. Several groups used a variety of clustering analyses to first organize the ASD related genes into networks, often leveraging their correlated expression across human brain development to group them into co-expression modules using WGCNA (Ben-David and Shifman, 2012; Parikshak et al., 2013), or philosophically similar approaches using additional data resources (Willsey et al., 2013; Chang et al., 2015). Resulting modules can be used for GO analyses or examined for enriched expression in particular developmental windows, brain regions, or cell types. It is worth noting here that it has long been recognized that one of the primary drivers of correlated gene expression across different brain regions is the consistent changes in proportions of different cell types (e.g., neurons and glia) across regions (Geschwind, 2000). Thus it is likely that many co-expression modules might correspond to genes enriched in a particular cell type. Our cell-type specific expression analysis (CSEA) approach (Doyle et al., 2008; Xu et al., 2014) or other datasets (Cahoy et al., 2008; Zhang et al., 2014) can be used to rapidly identify this. Regardless, in the above analyses, either co-expression or somewhat more inclusive human genetics criteria has been used to expand these ASD-related gene sets into larger modules. This allows for more genes to be included in these analyses, facilitating better network insights, though it is currently unclear if there is a particular cost in terms of a potentially inflated false positive rate associated with this expansion of gene sets.

However, in spite of the moderate differences in the precise ASD-related gene sets, differences in leveraged data resources, differences in the use of ‘top down’ or ‘bottom up’ methods and statistical approaches, some themes seem to be emerging regarding where ASD-related genes show enriched expression (**Table 2**). *First*, amongst the rare mutations that were highlighted in the recent exome studies, several groups have reported disproportionate expression in the mid fetal developing cortex and/or striatum (Parikshak et al., 2013; Willsey et al., 2013; Xu et al., 2014). Though there is some disagreement on the exact lamina that might be implicated (frankly, relatively few gene expression differences define distinct cortical lamina (Doyle et al., 2008; Dougherty et al., 2010; Xu et al., 2014) relative to the robust expression differences between cell types in other brain regions such as the cerebellum), many of these genes show relatively high expression in forebrain development. This is consistent with the long known roles in telencephalic development for at least two of the recently implicated genes (*TBR1* and *RELN*; Caviness and Sidman, 1973; Hevner et al., 2001), and suggest that mutations profoundly affecting forebrain development may have ASD as one (of perhaps many) deleterious consequences. This is consistent with the replicated finding that individuals with *de novo* loss-of-function mutations have lower IQ than other individuals with ASD (Samocha et al., 2014). *Second*, genes downregulated in human ASD postmortem transcriptomic studies (Voineagu et al., 2011; Gupta et al., 2014), and ASD candidate genes compiled prior to exome studies (Basu et al., 2009) seem to map most strongly to cortical interneurons, as well as a striatal cell type: medium spiny neurons (Xu et al., 2014). These findings suggest that perhaps there might be some shared abnormalities in cortical and striatal circuits across distinct genetic causes of ASD. In contrast, for example, none of the analyses have implicated cell types of the cerebellum, suggesting these are perhaps less commonly involved in ASD.

**Table 2:** Summary of findings regarding cell types, brain regions, or developmental windows with enriched expression of ASD genes.

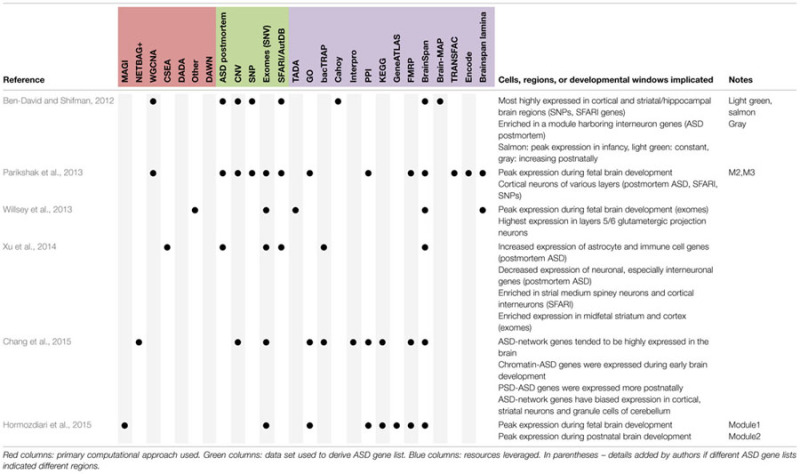

While overall both ‘top down’ and ‘bottom up’ discovery-driven approaches have highlighted potential circuits of interest in ASD, it is also clear that this disorder is not like retinopathies, which have a dominant signal in one or two cell types (minimum *p*-values < 10e^-20^). The significant, yet relatively modest statistical signal in ASD studies (minimum *p*-values around 10e^-3^ for medium spiny neurons or cortical interneurons) indicate there may be substantial heterogeneity in cellular mechanisms for the disorder, just as there is extensive heterogeneity in genetic mechanisms. Further, as many of these methods start from largely similar ASD-related gene lists, and leverage a small number of overlapping data resources, they do not truly represent independent replications. Thus, in the final section, we outline some of the challenges facing application of these approaches to ASD and present some examples of solutions and recommendations. The recommendations are not exhaustive and it is likely other elegant solutions exist as well. The challenges can be organized into three groups. *First*, how do we best identify and rule out alternative explanations that may also account for the relationships between these genes? How do we define the null hypothesis? And what are likely sources of false negatives? *Second*, how do we assess the reproducibility of a discovery-driven network analysis result? What constitutes a replication of one of these findings? *Finally*, how do we convert discovery-driven network-based insights into empirically testable hypotheses, and from there into informed treatments?

### Challenges Posed by Systems Biology Approaches using ASD-Related Genes

#### Challenge 1: Selecting the Correct Interpretation of a Network Analysis Result

**FIGURE 1:**
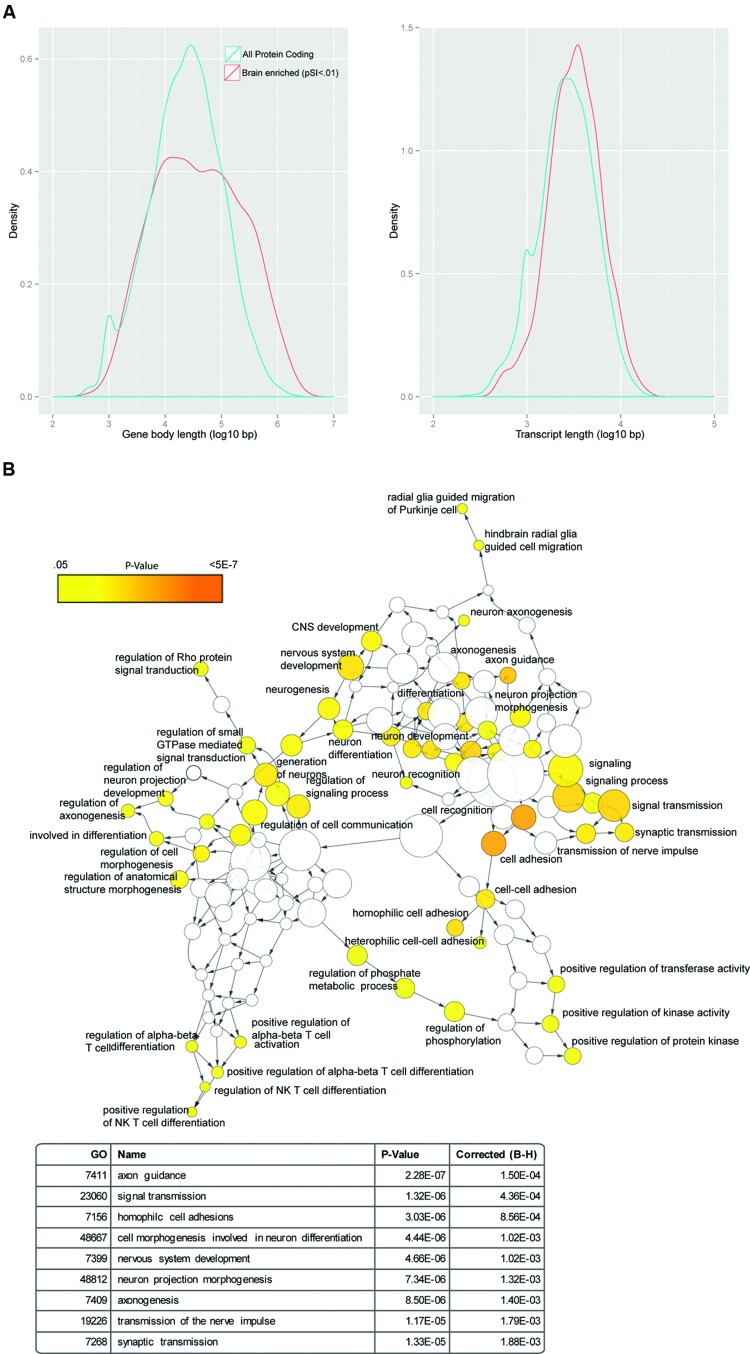
**Genes with enriched expression in the brain are long. (A)** Genes with enriched expression in the brain are longer both as mature transcripts (right) and in terms of gene body length (left), when compared to protein coding genes as a whole. **(B)** Random GWAS results show enrichment in a network of brain-related GO terms: using a uniform distribution between 0 and 1, random *p*-values were assigned to SNPs in the genome, and SNPs were mapped to genes using ANNOVAR. The SNP with the lowest *p*-value in a gene was used to determine the 500 most significant genes that were then used for a GO analysis and displayed as a network using BINGO. Dozens of categories related to CNS function were significant (examples shown in table at bottom).

**FIGURE 2:**
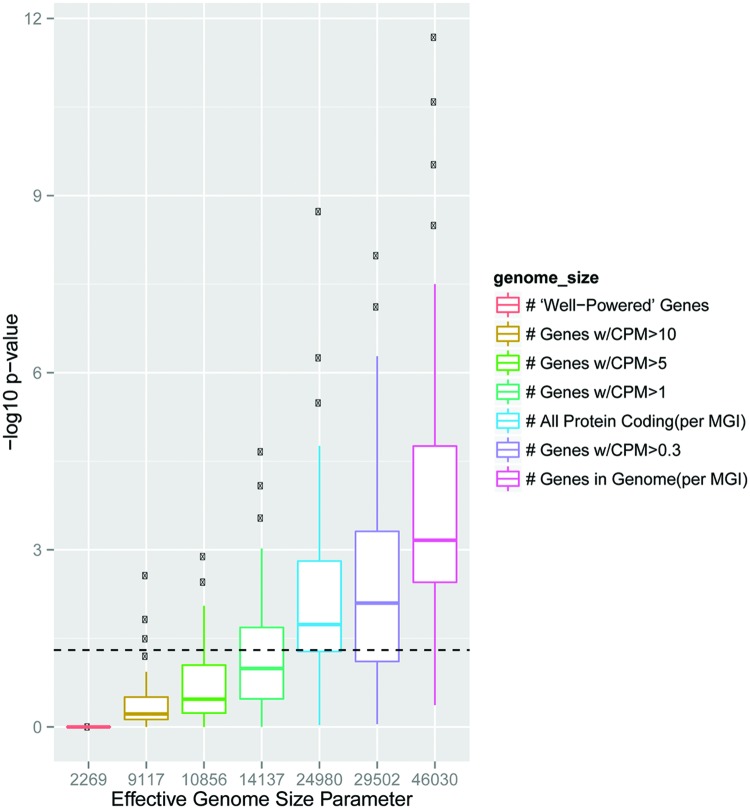
**Choice of effective genome size in GO analysis of transcriptomic data can substantially influence statistical results.** *Boxplots show distribution of -Log10 p-values for Fisher’s Exact Test overlapping the GO:007268, Synaptic transmission, with 100 different sampled lists at each effective genome size indicated. Dashed line is p = 0.05* Power to detect differential expression in RNAseq is influenced by total count number for a particular gene, and thus longer or higher expressed transcripts in a tissue are more likely to be found as significantly different (Bullard et al., 2010; Young et al., 2010). To determine if this could create spurious GO results from a brain transcriptomic experiment, we randomly sampled genes from the 10% most robustly detected genes in a brain RNAseq experiment (Ouwenga and Dougherty, 2015) to mimic results a 100 hypothetical differential expression experiments. If it was assumed these genes were randomly drawn without bias from the whole genome (*n* = 46030 genes) this consistently resulted in a statistically significant, but scientifically meaningless, enrichment in the GO category 0007268 (Synaptic Transmission). More conservative estimates which only include genes at least lowly expressed in the brain (CPM > 0.3 or CPM > 1) still frequently yield spurious overlap (*blue, purple*). Because of this, using an effective genome or ‘background gene set’ based on the transcripts which are well-powered for differential expression is recommended.

Networks are graphical descriptions of the relationships between the embedded entities. They provide the ability to display more numerous relationships than could be efficiently conveyed with words. However, a mind presented with such a large amount of data will rapidly organize it by drawing on examples from our own experience as researchers (idols of the cave yet again). Cortical development researchers might tend to migrate toward the cytoskeletal and Rho-GTPase genes, while physiologists may be most stimulated by the channel genes. One who has worked for many years with the transcriptional profiles of different cell classes in the brain, when looking at a network (or gene set), might have a bias to interpret it in terms of the cell types these genes are expressed in (i.e., one could view an ‘immune’ module in transcriptomic data as reflecting changes in the proportion of microglia in the tissue, rather than immune genes being upregulated in neurons). Thus, all investigators must be careful to recognize their individual biases for what they are and shield analytical approaches as best possible from them. In addition, there can be biases in the discovery methods and resources themselves that might create statistically significant results for scientifically insignificant reasons (**Figures 1** and **2**). Therefore, we also need to define as carefully as possible our null hypotheses and be attentive to circularity and alternative explanations.

**Key for both tables.**

**Approaches**

1. CSEA: cell specific expression analysis. A method to define sets of genes with enriched expression in particular samples – i.e., to create datasets that can serve as a “Gene Ontologies” for expression of genes in specific cell types. Applied initially to bacTRAP data.
2. DADA: disease aware disease-gene association: A method for prioritizing candidate genes within protein–protein interacting (or other networks) accounting for connectedness to other disease genes.
3. DAWN: detecting association within networks. A method leveraging gene expression data and genetic association scores to generate networks of related genes and prioritize additional candidate autism genes.
4. MAGI: merging affected genes into integrated networks. A method leveraging PPI and expression data to identify modules enriched in ASD genes. Can be used to prioritize additional candidate genes.
5. NETBAG: network based analysis of genetic associations. A method of constructing networks of related genes based on a variety of data sources including GO, KEGG, and PPI data.
6. WGCNA: weighted gene coexpression network analysis. A method of defining modules of genes with correlated expression patterns across samples.

**ASD geneset sources**

1. ASD postmortem: Microarray data comparing cortex and cerebellum of ASD patients and controls (Voineagu et al., 2011);
2. CNV: copy number variant studies;
3. SNP: single nucleotide polymorphism (common variant) studies;
4. Exomes/SNVs: single nucleotide variants (rare and *de novo*) studies;
5. SFARI/AutDB: a curated database of autism genes (initially AutDB), now hosted by Simons Foundation (Basu et al., 2009).

**Resources leveraged**

1. GOs: gene ontologies. Curated lists of functional annotations for all genes based on a defined hierarchical vocabulary. Accessed through a variety of portals across studies.
2. KEGG: Kyoto encyclopedia of genes and genomes. A curated collection of metabolic and signaling pathways, and associated genes.
3. Interpro: database of annotating the domains found in proteins.
4. PPI: protein–protein interaction data. Various sources across the studies (e.g., StringDB, Szklarczyk et al., 2015).
5. TADA: transmission and *de novo* association. A method for weighting genes by their likely contribution to ASD based on multiple sources of information (He et al., 2013). Here, used to refer to gene lists deriving from that method. Can also be used to prioritize genes, and is integrated with DAWN.
6. bacTRAP: gene expression profiles of ∼25 genetically defined cell populations in adult mice (Doyle et al., 2008).
7. Cahoy: gene expression profiles of the four major classes of cells in developing mouse brain (Cahoy et al., 2008).
8. GeneAtlas: a microarray study of dozens of distinct tissues in mouse and human (Su et al., 2004).
9. FMRP: the set of transcripts detected as binding the RNA binding protein FMRP in mouse brain (Darnell et al., 2011).
10. Brainspan: a collection of human brain transcriptomic data across multiple developmental time points and regions (Kang et al., 2011). Accessed through different portals in different studies.
11. Lamina: a collection of transcriptomic data using laser capture microdissection to harvest RNA from specific layers of developing human and mouse cortex (Miller et al., 2014).
12. BrainMap: a collection of human brain transcriptomic data from a single time point but across multiple regions from two individuals generate by Allen Institute as part of the Human Brain Atlas Microarray Survey.
13. Transfac: transcription factor database. A collection of sequence motifs known to bind specific transcription factors.
14. ENCODE: encyclopedia of DNA elements. A collection of many different types of data focused on identifying regions of genome covered by specific sets of epigenetic marks found on DNA in a range of tissues and cultured cell types.

##### Recommendation 1: define the null and rule out alternative explanations

Not all genes are equally likely to be implicated in genetic studies. A simple example is that longer genes will tend to, by nature of their size, overlap with more markers present on SNP microarrays, provide more bases that could have a *de novo* SNV and are more likely to be disrupted by a random CNV mutation. And of course, mutations are not randomly distributed: different regions of the genome, or even particular nucleotide contexts, have different rates of mutation (Krawczak et al., 1998; Michaelson et al., 2012; Samocha et al., 2014). Furthermore, transcript length, and potentially gene body size, bear some relationship to biological function. Notably, genes expressed in the nervous system tend to be longer in both regards (**Figure 1A**). For example, randomly sampling SNPs from the genome and mapping them to overlapping genes will result in an enrichment for brain-related GO categories (**Figure 1B**). Likewise in transcriptomic studies, the appropriate background ‘genome’ needs to be carefully defined (**Figure 2**). Transcripts clustering in modules or being differentially regulated in a particular tissue, by necessity must first be expressed in that tissue. Thus, the effective genome and genome size for statistical analysis of overlap should be restricted to those genes whose transcripts could have plausibly been identified in the analysis. As an example, both length and expression can come into play when considering overlaps with the known Fmrp-interacting RNAs (Darnell et al., 2011), as for potentially methodological reasons these tend to include long transcripts that are highly expressed in the brain. Therefore, the overlap of these RNAs with gene sets derived from either human genetic studies or potentially transcriptomic studies may reflect these primary features of the transcripts rather than a central role for Fmrp in the particular experiment (Ouwenga and Dougherty, 2015).

Overall, correcting for gene body length, transcript length, and brain expression level are challenging. For example, simply down-weighting GWAS results for those genes that are tagged by more SNPs, under the assumption that every gene in the genome is equally likely to contribute to disease, would be too conservative – long genes could legitimately be more vulnerable to mutation/polymorphism because of their length. And, an evolutionary argument could be made that genes requiring more careful regulation have evolved to be longer – permitting the presence of more potential regulatory sites (e.g., enhancers in the genome, or protein binding motifs in the RNA) to finely tune final protein levels. Indeed, genes that do not appear to tolerate heterozygous mutations in humans (Samocha et al., 2014), tend to be longer than the average gene. Thus, there is a risk that fully removing the influence of gene or transcript length in some analyses might be too conservative. Nonetheless, these are issues that should be explicitly addressed in analyses and chosen parameters should either be well-justified, or systematically varied to demonstrate robustness.

Therefore, an appropriate null for discovery-driven analyses of ASD-related genes should take these primary sequence features into account. A common approach is to conduct comparison analyses using sampled control sets of genes that share length, connectivity, or mutability with the ASD related genes (Willsey et al., 2013; Krumm et al., 2014; Chang et al., 2015). An additional control commonly used are genes actually detected as mutated (SNVs or CNVs) from control populations such as unaffected siblings, population databases of variation, or an unrelated disease (Pinto et al., 2010, 2014; Gilman et al., 2011; Chahrour et al., 2012; Parikshak et al., 2013; Krumm et al., 2014; Samocha et al., 2014; Chang et al., 2015; Hormozdiari et al., 2015). This controls for both the known biases highlighted above and any currently unrecognized biases in the ASD gene discovery methods.

#### Challenge 2: Independent Replication of a Network Analysis Result

One of the tenants of the scientific method is reproducibility. Experiments should be able to be reproduced by other labs and result in substantially identical findings. Furthermore, following the deductive tradition, tests of the same hypothesis using different methods should produce convergent results if the model is correct and the methods are robust. While discovery-driven analyses are typically insight- or hypothesis-generating rather than hypothesis-testing endeavors, reproducibility and replication are criteria that are still applicable.

##### Recommendation 2: parameter choices and code sharing

Just as in biological studies, where minor changes in the composition of a buffer can sometimes substantially alter biochemical findings, minor alterations in parameters in bioinformatic analyses can result in substantial differences in the results. For example, the choice of the effective genome size can dramatically influence p-values in analyses overlapping two gene sets with a Fisher’s Exact Test (**Figure 2**), and parameter choices in aligners have at times created misleading results such as an overestimation of RNA editing (Schrider et al., 2011). Frequently there may not be a strong *a priori* reason for choosing a particular parameter setting, which might lead to a ‘parameter placebo:’ an accidental or subconscious tuning of the parameter to produce the most striking results. To avoid this, key parameters can be varied systemically with the results presented in such a way that allows the reader to judge for themselves the robustness. For example, we were interested in overlapping sets of genes with ‘enriched’ expression in a particular cell type with ASD-related gene sets. We could rank genes from most enriched to least, but justifying a precise threshold was challenging. Uniquely expressed in these cells? In them, but in a few other populations as well (i.e., moderately enriched)? As there was no clear answer, we designed the analysis to systematically vary the parameter and present the results at multiple thresholds, with the most intuitive confidence given to overlaps that occurred significantly across some or all thresholds (Xu et al., 2014).

Thus the researcher’s choice for how parameters are set (or the range of values tested) needs to be well-justified. Code for conducting the entire analysis should be made available on request, or perhaps even hosted in its entirety on a public forum. However, to enable this, the discovery of a bug in code that has been made available should be treated as an opportunity to raise the quality of the scientific analyses as a whole, rather than as an opportunity to cast stones at a competing lab.

##### Recommendation 3: replication replication replication…

It is a common experience at the bench – the first replicate of an experimental series that matches predictions perfectly or that hints at exciting new biology. And then the second replicate that does not match the first, and then third, fourth, fifth, until it becomes apparent that the first experiment was the outlier, whether due to some technical mishap or simple winner’s curse. At the bench one has the (dubious) luxury of being able to repeat an experiment as many times as cost and time constraints allow to convince ourselves of the reproducibility of an outcome. However, in systems biology, there is often only one starting candidate disease gene set with which to seed your network. And largely only one GO or Brainspan resource to compare it with. One can rerun the analysis to make sure the same result occurs (analogous to a ‘technical replicate’ at the bench), but this is not as reassuring as a true independent biological replicate would be. In general, replications of transcriptome analyses in independent samples have been difficult historically, and these discrepancies have been attributed to variations in study design, processing of samples, and/or computational methods (Gudjonsson et al., 2010; Suárez-Fariñas et al., 2010). Thus, assessing the reproducibility of a bioinformatic analysis is inherently challenging.

A fundamental question that must be faced is whether the inability to reproduce is due to systemic variations, such as those previously suggested, or due to failure to capture true biological signatures. A major obstacle for these studies is the difficulty in amassing large sample sizes and unfortunately, this issue is seldom addressed. Network construction is typically achieved by conducting some type of similarity or correlation tests across pairs of genes/proteins. Inadequate sample size can produce seemingly promising networks with strong community structure due to the clustering of false-positive correlations. Unfortunately, significant correlation thresholds are not one-size-fits-all and vary between datasets due to sample size, heterogeneity of samples and other factors. For this reason, we strongly recommend the use of a rigorous method for determining an appropriate threshold for edge placement during the construction of networks. For example, permutation trials provide a simple and robust method for determining appropriate correlation thresholds. For each trial, the data values for each gene are permuted across individuals, thereby retaining all of the properties of each gene except for inherent correlations with other genes. After running an adequate number of trials, e.g., 1000 trials, the highest correlation values computed across the uncorrelated permuted data can be used to determine a threshold with a desired *p*-value.

Assuming *bona fide* network construction, there are at least three, albeit imperfect, options for replication. *First*, borrowing from machine learning or human genetics studies, the starting ASD-related gene sets could be broken into artificial ‘discovery’ and ‘replication’ subsets, or even K subsets, so some form of K-fold cross-validation of the results could be conducted (assuming adequate sample size). Then at least the robustness of the results with regards to sample selection could be assessed (Refaeilzadeh et al., 2009). *Second*, comparisons of results with those identified by independent groups using distinct analytical approaches may yield strong evidence of biological validity. To an extent, it is very reassuring that multiple groups have drawn fairly similar conclusions when applying these approaches to ASD (**Tables 1** and **2**), though of course these are not true replications because they are not independent – as they draw on similar comparison data resources (i.e., regardless of whether GO is accessed through DAVID, Panther, BinGO, GOrilla or other portal, the gene sets are largely identical). Further, the Brainspan, bacTRAP, and Cahoy datasets have seen similar widespread use (Cahoy et al., 2008; Doyle et al., 2008; Kang et al., 2011). Thus, it will be even more reassuring if similar results about these ASD-related gene sets hold when additional comparison data resources come online, such as single cell expression studies (e.g., Zeisel et al., 2015). The *third* option is replication through the increasing size of the ASD-related gene sets through time. In this regard it is reassuring that many of the patterns seen in the early Capstone analyses (e.g., neurite morphogenesis/cytoskeletal elements) have been reproduced in later discovered gene sets (**Table 1**).

#### Challenge 3: Converting Discovery-Driven Insights into Empirically Testable Hypotheses

How does one test a network result functionally? The recent application of a variety of these inductive discovery-driven approaches to ASD-related genes have highlighted potential molecular gene sets or cell types common to different genetic causes of ASD (**Tables 1** and **2**). As gene discovery and *post hoc* analyses are expected to continue apace, a key challenge is the conversion of these insights into clearly stated hypotheses from which we can *deductively* define a set of empirically testable predictions. This is not a straightforward endeavor – as a key facet of any good hypothesis is that it be falsifiable, and it is not clear that is the case with the insights emerging from capstone analyses. Assuming there are no artifacts in the analysis, how does one falsify the hypothesis that chromatin modifiers are important in ASD? If, following the discovery of many more ASD risk genes in the next rounds of sequencing there is no longer a significant enrichment of this class of genes, does that mean the chromatin modifiers are now unimportant? One could argue no. Because for that small subset of ASD cases who carry a mutation in a chromatin modifier like *Chd8*, chromatin modifiers still play an important role. Rather, it would argue that chromatin modifying genes play a role, but only in rare cases. Thus, the implications of the insights garnered from a properly conducted discovery-driven analysis can change in scale, but never really go away. Only if the assumptions of the analysis itself change (e.g., *Chd8* turns out not to be a chromatin modifier) can the insight be falsified.

Yet, sometimes discovery-driven analyses can lead to the generation of falsifiable hypothesis. In a simple example, one could mutate the chromatin modifying function of *Chd8* or other candidates and measure whether that phenocopies complete loss of function in a model system. Other predictions can also be made about increased ASD risk or shared biological functions for genes that share many edges in a network. Below we highlight some experiments and suggest others that might meet this challenge.

##### Recommendation 4: testing network predictions with human genetics

The networks described in several of the recent analyses cited above by design both included genes confidently associated with ASD, and included genes that were either less confidently associated from human genetics, or were implicated by ‘guilt-by-association’ (Quackenbush, 2003) in *post hoc* analyses: e.g., that were perhaps co-expressed, co-annotated (GO), co-published (Text mining), or co-immunoprecipitated (PPI) with the more confidently associated genes. One prediction of these networks might be that mutations or polymorphism in these guilt-by-association genes will also cause or contribute to ASD risk. This concept can be tested informatically (e.g., looking for an increased common variant risk near such genes, though controlling appropriately for gene length, etc.; Ben-David and Shifman, 2012; Parikshak et al., 2013). More directly, O’Roak et al. (2012a), tested their network’s prediction by targeted resequencing of such genes in a large cohort of ASD patients, demonstrating novel statistical association for several of them, and showing the utility of the initial network analyses. Thus, these discovery-driven analyses can successfully serve to direct new studies in human genetics and additional studies may assist in more rapid identification of additional causative genes. This approach will continue to be useful for a few years, though eventually it is likely to be supplanted as sequencing costs decrease and targeted analyses are replaced by routine exome or whole genome sequencing. Likewise, inclusion in a particular gene set has been used to reweight the probability that a variant of unknown significance should be considered pathogenic (e.g., with the TADA algorithm De Rubeis et al., 2014), though the most compelling evidence continues to be the presence of recurrent mutations in cases of ASD.

##### Recommendation 5: phenotypic clustering in man and models

Another apparent feature of these analyses is that genes in the networks are somewhat clustered by function. Thus, these networks may be making testable predictions regarding the shared function of closely connected genes. If the genes do indeed share some function at the molecular or cellular level, then the prediction is that genes that are closer in the network will be closer in their consequences in cell models, animal models, or potentially even patient symptoms.

A point of caution is that failing to identify a significant similar phenotype or any phenotype at all when investigating a set of genes highlighted by a network analysis does not necessarily reject that hypothesis that the genes share some function. There are a myriad of possible phenotypes to evaluate, and all cannot be exhaustively tested. However, considering the information used to create the network and the resulting gene set can provide limits to the scope of phenotypes to test and prioritize primary outcomes of interest. And, at least the hypothesis that these genes have shared impact on those particular phenotypes does become testable and falsifiable.

A second point of caution is that the novelty of these predictions depends on whether functional data (e.g., PPI), or functional annotations (GO) were not used to build the networks in the first place – a caveat that cannot be taken lightly. Otherwise the network is not really making new predictions, just redisplaying known relationships in a different form. Likewise, there are no generally enforced standards for how these networks are displayed, and in some cases the authors may have selected the presentation of the nodes that maximally illustrates the functional clustering they would like to discuss. Overall, care must be taken to assure that circular logic does not creep into the conclusions drawn from these analyses.

**FIGURE 3:**
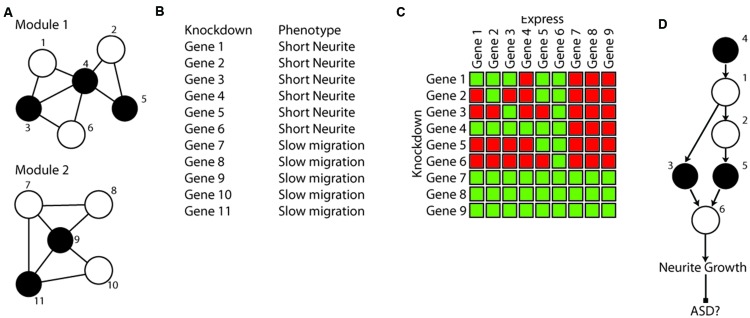
**Hypothetical example of phenotypical clustering and epistasis analysis in a culture model. (A)** A hypothetical network constructed with five ASD genes (black) has resulted in two modules of 5–6 genes that are connected by expression and PPI data that include both ASD genes and tightly connected genes (white) not yet implicated in ASD. **(B)** The network result leads to a hypothesis that genes that are in the same module regulate the same phenotype. This is tested using single gene knockdown in iPSC derived neurons followed by high content imaging of neuronal morphology and behavior. Knockdown of the members of the two modules results in distinct cellular phenotypes, consistent with them potentially representing two distinct mechanisms of developing ASD (potentially two subtypes requiring different treatments). In this hypothetical example the tightly connected genes show the same phenotype. **(C)** Epistasis analysis for neurite length is used to test the hypothesis that all genes in module 1 are in the same pathway regulating neurite growth and are distinct from genes in module 2. Single gene knockdowns of all genes are transiently transfected with constructs expressing each individual gene. Green squares are normal neurite length, red squares are shortened neurites. These hypothetical results suggest several conclusions: (1) negative control genes from module 2 can’t rescue short neurites, again indicating they are not in this genetic pathway. (2) As a control, expression of each gene in module 1 can rescue (complement) its own phenotype. (3) The pattern of complementation can be used to infer the functional relationship between the genes **(D)**. For example, gene 4 can be rescued by any other gene in the module, suggesting it must be before the others in the pathway, while gene 6 can rescue all others, but cannot be rescued by any of them, indicating it must be last. **(D)** The resulting pathway from the genetic analysis of neurite length. This result indicates that the original module did indeed represent a set of genes that regulate the same neurobiological phenomena. If indeed the shortened neurites lead to ASD, this also suggests that treatments targeting gene 6 (even though it was not itself an ASD gene), may be effective at treating individuals with ASD who have mutations in genes 3, 4, or 5.

Nonetheless, a variety of methods exist which could test any novel predictions regarding shared impact on phenotype. Deeply phenotyped sets of patients (such as the Simons collection; Fischbach and Lord, 2010) could be studied to determine whether individuals with mutations in genes that are closely spaced in the network share more clinical features. Specific mutations could be isolated or introduced in IPSC derived neural cells and their consequences studied with data rich methods such as hi-content imaging or RNAseq, with the prediction that there will be more similar phenotypic consequences for genes that are closer in the network (**Figure 3**). However, cultured cells have limitations in terms of the cell types that can be generated. They also have a very limited behavioral repertoire. Thus, it is our opinion that there is also a strong need to study the commonalities in behavioral disruptions across a variety of mice modeling these mutations. Though mouse behaviors are not meant to be perfect proxies for human symptoms, behaviors are highly sensitive readouts of the functions of particular CNS circuits in an intact organism. Shared behavioral disruptions across these can indicate shared circuit level disruptions, and particularly cell-type predictions (Parikshak et al., 2013; Willsey et al., 2013; Xu et al., 2014; Chang et al., 2015) might be best tested in the context of a complex nervous system. Cre-Lox and optogenetic technologies in particular provide the opportunity to explicitly test shared contributions of particular circuits downstream of a genetic lesion. There is also a clear need for a more systematic approach to behavioral phenotyping, as the current one-lab-evaluates-one-model approach, often in different genetic backgrounds, makes careful and systematic *post hoc* comparisons across models nearly impossible.

##### Recommendation 6: epistasis analysis in models and mice

Genetic formalism has a lot to offer in the context of testing these networks. Genes that are closer in the networks (or perhaps co-expressed in the same cell type), may be in the same functional pathway. Studying compound mutations in humans might be informative (Pinto et al., 2014; Krumm et al., 2015). Indeed, examination of >100 million medical records has been used to test for epistatic effects of combinations of rare Mendelian diagnoses on risk of developing comorbid complex disease traits (Blair et al., 2013). But with currently available sample sizes for exome-sequenced cases, multiple ASD-related rare variants are unlikely to occur in the same individual frequently enough for formal testing of specific pairs of ASD-related genes. However, both in animals and in cell lines it is straightforward to make compound mutations and introduce rescue constructs with modern genome editing technologies. Thus, not only can we test whether network-associated genes have similar phenotypes (**Figures 3A,B**), we can also test whether any gene is in the same pathway and if it is dominant, complementary or suppressive of others (**Figures 3C,D**). Again, this could leverage both cell lines and mouse models for their relative strengths of either throughput or complexity.

Finally, all recommendations 4–6 might also provide the opportunity to subtype ASD cases into functionally distinct categories based on their molecular causes or cellular consequences – separate categories which may indeed be most amenable to different treatment strategies or that warrant stratification during clinical trials. Already results from exome studies are being used to define new subtypes of ASD starting from knowledge of the implicated gene (Bernier et al., 2014). Understanding commonalties in different subtypes of patients might be key to identifying routes to treatments for each.

## Conclusion: Building New Cornerstones from Old Capstones

Over the last few years, discovery-driven bioinformatics analyses of ASD-related genes have moved from final figure capstone analyses to stand alone manuscripts. In architecture, the capstone is the coping, the final layer of finer, flat stone on the top of a wall of a that is somewhat functional (e.g., to end the structure, to protect from weather) but also somewhat decorative. Meanwhile, the cornerstones, classically, are the first stones placed in a new building – the seeds from which new buildings arise. Thus, the time has come to push these systems biology analyses away from capstones and toward cornerstones: studies from which we can derive empirically testable theories regarding commonalities of mechanism(s) for the diverse genetic risk factors contributing to ASD. The overall challenge now is to define criteria with which to systematically evaluate these discovery-driven insights, and to generate falsifiable hypotheses from these ideas. The hypotheses that survive rigorous empirical testing have the potential to become the foundations of new edifices rising toward ASD treatments.

## Conflict of Interest Statement

Joseph D. Dougherty has received licensing fees related to the bacTRAP technology. The other authors declare they have no financial or commercial relationships that could be construed as a potential conflict of interest.

## References

[B1] Anney R. Klei L. Pinto D. Almeida J. Bacchelli E. Baird G. (2012). Individual common variants exert weak effects on the risk for autism spectrum disorders. *Hum. Mol. Genet.* 21 4781–4792. 10.1093/hmg/dds301 22843504 PMC3471395

[B2] Ardlie K. G. Deluca D. S. Segrè A. V. Sullivan T. J. Young T. R. Gelfand E. T. (2015). The Genotype-tissue expression (GTEx) pilot analysis: multitissue gene regulation in humans. *Science* 348 648–660. 10.1126/science.1262110 PMC4547484 25954001

[B3] Ashburner M. Ball C. A. Blake J. A. Botstein D. Butler H. Cherry J. M. (2000). Gene ontology: tool for the unification of biology. *Nat. Genet.* 25 25–29. 10.1038/75556 10802651 PMC3037419

[B4] Bacon F. (1620). *The New Organan.* Available at: www.earlymoderntexts.com

[B5] Basu S. N. Kollu R. Banerjee-Basu S. (2009). AutDB: a gene reference resource for autism research. *Nucleic Acids Res.* 37 D832–D836. 10.1093/nar/gkn835 19015121 PMC2686502

[B6] Ben-David E. Shifman S. (2012). Networks of neuronal genes affected by common and rare variants in autism spectrum disorders. *PLoS Genet.* 8:e1002556 10.1371/journal.pgen.1002556 PMC3297570 22412387

[B7] Bernier R. Golzio C. Xiong B. Stessman H. A. Coe B. P. Penn O. (2014). Disruptive CHD8 mutations define a subtype of autism early in development. *Cell* 158 263–276. 10.1016/j.cell.2014.06.017 PMC4136921 24998929

[B8] Betancur C. (2011). Etiological heterogeneity in autism spectrum disorders: more than 100 genetic and genomic disorders and still counting. *Brain Res.* 1380 42–77. 10.1016/j.brainres.2010.11.078 21129364

[B9] Blair D. R. Lyttle C. S. Mortensen J. M. Bearden C. F. Jensen A. B. Khiabanian H. (2013). A nondegenerate code of deleterious variants in mendelian loci contributes to complex disease risk. *Cell* 155 70–80. 10.1016/j.cell.2013.08.030 24074861 PMC3844554

[B10] Bucan M. Abrahams B. S. Wang K. Glessner J. T. Herman E. I. Sonnenblick L. I. (2009). Genome-wide analyses of exonic copy number variants in a family-based study point to novel autism susceptibility genes. *PLoS Genet.* 5:e1000536 10.1371/journal.pgen.1000536 PMC2695001 19557195

[B11] Bullard J. H. Purdom E. Hansen K. D. Dudoit S. (2010). Evaluation of statistical methods for normalization and differential expression in mRNA-Seq experiments. *BMC Bioinformatics* 11:94 10.1186/1471-2105-11-94 PMC2838869 20167110

[B12] Cahoy J. D. Emery B. Kaushal A. Foo L. C. Zamanian J. L. Christopherson K. S. (2008). A transcriptome database for astrocytes, neurons, and oligodendrocytes: a new resource for understanding brain development and function. *J. Neurosci.* 28 264–278. 10.1523/JNEUROSCI.4178-07.2008 18171944 PMC6671143

[B13] Caviness V. S. Sidman R. L. (1973). Retrohippocampal, hippocampal and related structures of the forebrain in the reeler mutant mouse. *J. Comp. Neurol.* 147 235–253. 10.1002/cne.901470206 4682775

[B14] Chahrour M. H. Yu T. W. Lim E. T. Ataman B. Coulter M. E. Hill R. S. (2012). Whole-exome sequencing and homozygosity analysis implicate depolarization-regulated neuronal genes in autism. *PLoS Genet.* 8:e1002635 10.1371/journal.pgen.1002635 PMC3325173 22511880

[B15] Chang J. Gilman S. R. Chiang A. H. Sanders S. J. Vitkup D. (2015). Genotype to phenotype relationships in autism spectrum disorders. *Nat. Neurosci.* 18 191–198. 10.1038/nn.3907 25531569 PMC4397214

[B16] Darnell J. C. Van Driesche S. J. Zhang C. Hung K. Y. S. Mele A. Fraser C. E. (2011). FMRP stalls ribosomal translocation on mRNAs linked to synaptic function and autism. *Cell* 146 247–261. 10.1016/j.cell.2011.06.013 PMC3232425 21784246

[B17] Deng P.-Y. Rotman Z. Blundon J. A. Cho Y. Cui J. Cavalli V. (2013). FMRP regulates neurotransmitter release and synaptic information transmission by modulating action potential duration via BK channels. *Neuron* 77 696–711. 10.1016/j.neuron.2012.12.018 23439122 PMC3584349

[B18] De Rubeis S. He X. Goldberg A. P. Poultney C. S. Samocha K. Ercument Cicek A. (2014). Synaptic, transcriptional and chromatin genes disrupted in autism. *Nature* 515 209–215. 10.1038/nature13772 25363760 PMC4402723

[B19] Dougherty J. D. Schmidt E. F. Nakajima M. Heintz N. (2010). Analytical approaches to RNA profiling data for the identification of genes enriched in specific cells. *Nucleic Acids Res.* 38 4218–4230. 10.1093/nar/gkq130 20308160 PMC2910036

[B20] Doyle J. P. Dougherty J. D. Heiman M. Schmidt E. F. Stevens T. R. Ma G. (2008). Application of a translational profiling approach for the comparative analysis of CNS cell types. *Cell* 135 749–762. 10.1016/j.cell.2008.10.029 19013282 PMC2763427

[B21] Fischbach G. D. Lord C. (2010). The simons simplex collection: a resource for identification of autism genetic risk factors. *Neuron* 68 192–195. 10.1016/j.neuron.2010.10.006 20955926

[B22] Gai X. Xie H. M. Perin J. C. Takahashi N. Murphy K. Wenocur A. S. (2012). Rare structural variation of synapse and neurotransmission genes in autism. *Mol. Psychiatry* 17 402–411. 10.1038/mp.2011.10 21358714 PMC3314176

[B23] Gaugler T. Klei L. Sanders S. J. Bodea C. A. Goldberg A. P. Lee A. B. (2014). Most genetic risk for autism resides with common variation. *Nat. Genet.* 46 881–885. 10.1038/ng.3039 25038753 PMC4137411

[B24] Geschwind D. H. (2000). Mice, microarrays, and the genetic diversity of the brain. *Proc. Natl. Acad. Sci. U.S.A.* 97 10676–10678. 10.1073/pnas.97.20.10676 11005850 PMC34042

[B25] Gilman S. R. Iossifov I. Levy D. Ronemus M. Wigler M. Vitkup D. (2011). Rare de novo variants associated with autism implicate a large functional network of genes involved in formation and function of synapses. *Neuron* 70 898–907. 10.1016/j.neuron.2011.05.021 21658583 PMC3607702

[B26] Glessner J. T. Wang K. Cai G. Korvatska O. Kim C. E. Wood S. (2009). Autism genome-wide copy number variation reveals ubiquitin and neuronal genes. *Nature* 459 569–573. 10.1038/nature07953 19404257 PMC2925224

[B27] Gudjonsson J. E. Ding J. Johnston A. Tejasvi T. Guzman A. M. Nair R. P. (2010). Assessment of the psoriatic transcriptome in a large sample: additional regulated genes and comparisons with in vitro models. *J. Invest. Dermatol.* 130 1829–1840. 10.1038/jid.2010.36 20220767 PMC3128718

[B28] Gupta S. Ellis S. E. Ashar F. N. Moes A. Bader J. S. Zhan J. (2014). Transcriptome analysis reveals dysregulation of innate immune response genes and neuronal activity-dependent genes in autism. *Nat. Commun.* 5:5748 10.1038/ncomms6748 PMC4270294 25494366

[B29] Hall A. Lalli G. (2010). Rho and Ras GTPases in axon growth, guidance, and branching. *Cold Spring Harb. Perspect. Biol.* 2:a001818 10.1101/cshperspect.a001818 PMC2828272 20182621

[B30] He X. Sanders S. J. Liu L. De Rubeis S. Lim E. T. Sutcliffe J. S. (2013). Integrated model of de novo and inherited genetic variants yields greater power to identify risk genes. *PLoS Genet.* 9:e1003671 10.1371/journal.pgen.1003671 PMC3744441 23966865

[B31] Hevner R. F. Shi L. Justice N. Hsueh Y.-P. Sheng M. Smiga S. (2001). Tbr1 regulates differentiation of the preplate and layer 6. *Neuron* 29 353–366. 10.1016/S0896-6273(01)00211-2 11239428

[B32] Hormozdiari F. Penn O. Borenstein E. Eichler E. E. (2015). The discovery of integrated gene networks for autism and related disorders. *Genome Res.* 25 142–154. 10.1101/gr.178855.114 25378250 PMC4317170

[B33] Huitema B. E. (2005). “Analysis of covariance,” in *Encyclopedia of Statistics in Behavioral Science* eds Everitt S. Howell D. (Chichester: John Wiley & Sons, Ltd).

[B34] Iossifov I. O’Roak B. J. Sanders S. J. Ronemus M. Krumm N. Levy D. (2014). The contribution of de novo coding mutations to autism spectrum disorder. *Nature* 515 216–221. 10.1038/nature13908 25363768 PMC4313871

[B35] Kang H. J. Kawasawa Y. I. Cheng F. Zhu Y. Xu X. Li M. (2011). Spatio-temporal transcriptome of the human brain. *Nature* 478 483–489. 10.1038/nature10523 22031440 PMC3566780

[B36] Kell D. B. Oliver S. G. (2004). Here is the evidence, now what is the hypothesis? The complementary roles of inductive and hypothesis-driven science in the post-genomic era. *Bioessays* 26 99–105. 10.1002/bies.10385 14696046

[B37] Klei L. Sanders S. J. Murtha M. T. Hus V. Lowe J. K. Willsey A. J. (2012). Common genetic variants, acting additively, are a major source of risk for autism. *Mol. Autism* 3:9 10.1186/2040-2392-3-9 PMC3579743 23067556

[B38] Krawczak M. Ball E. V. Cooper D. N. (1998). Neighboring-nucleotide effects on the rates of germ-line single-base-pair substitution in human genes. *Am. J. Hum. Genet.* 63 474–488. 10.1086/301965 9683596 PMC1377306

[B39] Krumm N. O’Roak B. J. Shendure J. Eichler E. E. (2014). A de novo convergence of autism genetics and molecular neuroscience. *Trends Neurosci.* 37 95–105. 10.1016/j.tins.2013.11.005 24387789 PMC4077788

[B40] Krumm N. Turner T. N. Baker C. Vives L. Mohajeri K. Witherspoon K. (2015). Excess of rare, inherited truncating mutations in autism. *Nat. Genet.* 47 582–588. 10.1038/ng.3303 25961944 PMC4449286

[B41] Lage K. Karlberg O. Størling M. Ólason Í. Pedersen G. Rigina O. (2007). A human phenome-interactome network of protein complexes implicated in genetic disorders. *Nat. Biotechnol.* 25 309–316. 10.1038/nbt1295 17344885

[B42] Levy D. Ronemus M. Yamrom B. Lee Y. Leotta A. Kendall J. (2011). Rare de novo and transmitted copy-number variation in autistic spectrum disorders. *Neuron* 70 886–897. 10.1016/j.neuron.2011.05.015 21658582

[B43] Ma D. Salyakina D. Jaworski J. M. Konidari I. Whitehead P. L. Andersen A. N. (2009). A genome-wide association study of autism reveals a common novel risk locus at 5p14.1. *Ann. Hum. Genet.* 73 263–273. 10.1111/j.1469-1809.2009.00523.x 19456320 PMC2918410

[B44] Malhotra D. Sebat J. (2012). CNVs: harbingers of a rare variant revolution in psychiatric genetics. *Cell* 148 1223–1241. 10.1016/j.cell.2012.02.039 22424231 PMC3351385

[B45] Marshall C. R. Noor A. Vincent J. B. Lionel A. C. Feuk L. Skaug J. (2008). Structural variation of chromosomes in autism spectrum disorder. *Am. J. Hum. Genet.* 82 477–488. 10.1016/j.ajhg.2007.12.009 18252227 PMC2426913

[B46] Michaelson J. J. Shi Y. Gujral M. Zheng H. Malhotra D. Jin X. (2012). Whole genome sequencing in autism identifies hotspots for de novo germline mutation. *Cell* 151 1431–1442. 10.1016/j.cell.2012.11.019 23260136 PMC3712641

[B47] Miller J. A. Ding S.-L. Sunkin S. M. Smith K. A. Ng L. Szafer A. (2014). Transcriptional landscape of the prenatal human brain. *Nature* 508 199–206. 10.1038/nature13185 24695229 PMC4105188

[B48] Neale B. M. Kou Y. Liu L. Ma’ayan A. Samocha K. E. Sabo A. (2012). Patterns and rates of exonic de novo mutations in autism spectrum disorders. *Nature* 485 242–245. 10.1038/nature11011 22495311 PMC3613847

[B49] O’Roak B. J. Vives L. Fu W. Egertson J. D. Stanaway I. B. Phelps I. G. (2012a). Multiplex targeted sequencing identifies recurrently mutated genes in autism spectrum disorders. *Science* 338 1619–1622. 10.1126/science.1227764 23160955 PMC3528801

[B50] O’Roak B. J. Vives L. Girirajan S. Karakoc E. Krumm N. Coe B. P. (2012b). Sporadic autism exomes reveal a highly interconnected protein network of de novo mutations. *Nature* 485 246–250. 10.1038/nature10989 22495309 PMC3350576

[B51] Ouwenga R. L. Dougherty J. (2015). Fmrp targets or not: long, highly brain-expressed genes tend to be implicated in autism and brain disorders. *Mol. Autism* 6:16 10.1186/s13229-015-0008-1 PMC4363463 25789151

[B52] Parikshak N. N. Luo R. Zhang A. Won H. Lowe J. K. Chandran V. (2013). Integrative functional genomic analyses implicate specific molecular pathways and circuits in autism. *Cell* 155 1008–1021. 10.1016/j.cell.2013.10.031 24267887 PMC3934107

[B53] Peyron C. Faraco J. Rogers W. Ripley B. Overeem S. Charnay Y. (2000). A mutation in a case of early onset narcolepsy and a generalized absence of hypocretin peptides in human narcoleptic brains. *Nat. Med.* 6 991–997. 10.1038/79690 10973318

[B54] Pinto D. Delaby E. Merico D. Barbosa M. Merikangas A. Klei L. (2014). Convergence of genes and cellular pathways dysregulated in autism spectrum disorders. *Am. J. Hum. Genet.* 94 677–694. 10.1016/j.ajhg.2014.03.018 24768552 PMC4067558

[B55] Pinto D. Pagnamenta A. T. Klei L. Anney R. Merico D. Regan R. (2010). Functional impact of global rare copy number variation in autism spectrum disorders. *Nature* 466 368–372. 10.1038/nature09146 20531469 PMC3021798

[B56] Pritchard J. K. Stephens M. Donnelly P. (2000). Inference of population structure using multilocus genotype data. *Genetics* 155 945–959. 10835412 10.1093/genetics/155.2.945 PMC1461096

[B57] Purcell S. Neale B. Todd-Brown K. Thomas L. Ferreira M. A. R. Bender D. (2007). PLINK: a tool set for whole-genome association and population-based linkage analyses. *Am. J. Hum. Genet.* 81 559–575. 10.1086/519795 17701901 PMC1950838

[B58] Quackenbush J. (2003). Genomics. Microarrays–guilt by association. *Science* 302 240–241. 10.1126/science.1090887 14551426

[B59] Reed D. A. Bajcsy R. Fernandez M. A. Griffiths J.-M. Mott R. D. Dongarra J. (2005). *Computational Science: Ensuring America’s Competitivenes.* Arlington, VA: National Coordination Office for Information Technology Research & Development.

[B60] Refaeilzadeh P. Tang L. Liu H. (2009). “Cross-validation,” in *Encyclopedia of Database Systems* eds Liu L. Özsu M. T. (New York, NY: Springer) 532–538.

[B61] Rossin E. J. Lage K. Raychaudhuri S. Xavier R. J. Tatar D. Benita Y. (2011). Proteins encoded in genomic regions associated with immune-mediated disease physically interact and suggest underlying biology. *PLoS Genet.* 7:e1001273 10.1371/journal.pgen.1001273 PMC3020935 21249183

[B62] Samocha K. E. Robinson E. B. Sanders S. J. Stevens C. Sabo A. McGrath L. M. (2014). A framework for the interpretation of de novo mutation in human disease. *Nat. Genet.* 46 944–950. 10.1038/ng.3050 25086666 PMC4222185

[B63] Sanders S. J. Ercan-Sencicek A. G. Hus V. Luo R. Murtha M. T. Moreno-De-Luca D. (2011). Multiple recurrent de novo CNVs, including duplications of the 7q11.*23* Williams syndrome region, are strongly associated with autism. *Neuron* 70 863–885. 10.1016/j.neuron.2011.05.002 21658581 PMC3939065

[B64] Sanders S. J. Murtha M. T. Gupta A. R. Murdoch J. D. Raubeson M. J. Willsey A. J. (2012). De novo mutations revealed by whole-exome sequencing are strongly associated with autism. *Nature* 485 237–241. 10.1038/nature10945 22495306 PMC3667984

[B65] Schrider D. R. Gout J.-F. Hahn M. W. (2011). Very Few RNA and DNA sequence differences in the human transcriptome. *PLoS ONE* 6:e25842 10.1371/journal.pone.0025842 PMC3192132 22022455

[B66] Sebat J. Lakshmi B. Malhotra D. Troge J. Lese-Martin C. Walsh T. (2007). Strong association of de novo copy number mutations with autism. *Science* 316 445–449. 10.1126/science.1138659 17363630 PMC2993504

[B67] Su A. I. Wiltshire T. Batalov S. Lapp H. Ching K. A. Block D. (2004). A gene atlas of the mouse and human protein-encoding transcriptomes. *Proc. Natl. Acad. Sci. U.S.A.* 101 6062–6067. 10.1073/pnas.0400782101 15075390 PMC395923

[B68] Suárez-Fariñas M. Lowes M. A. Zaba L. C. Krueger J. G. (2010). Evaluation of the psoriasis transcriptome across different studies by gene set enrichment analysis (gsea). *PLoS ONE* 5:e10247 10.1371/journal.pone.0010247 PMC2857878 20422035

[B69] Szklarczyk D. Franceschini A. Wyder S. Forslund K. Heller D. Huerta-Cepas J. (2015). STRING v10: protein-protein interaction networks, integrated over the tree of life. *Nucleic Acids Res.* 43 D447–D452. 10.1093/nar/gku1003 25352553 PMC4383874

[B70] Voineagu I. Wang X. Johnston P. Lowe J. K. Tian Y. Horvath S. (2011). Transcriptomic analysis of autistic brain reveals convergent molecular pathology. *Nature* 474 380–384. 10.1038/nature10110 21614001 PMC3607626

[B71] Wang K. Zhang H. Ma D. Bucan M. Glessner J. T. Abrahams B. S. (2009). Common genetic variants on 5p14.1 associate with autism spectrum disorders. *Nature* 459 528–533. 10.1038/nature07999 19404256 PMC2943511

[B72] Weiss L. A. Arking D. E. Gene Discovery Project of Johns Hopkins and the Autism Consortium Daly M. J. Chakravarti A. (2009). A genome-wide linkage and association scan reveals novel loci for autism. *Nature* 461 802–808. 10.1038/nature08490 19812673 PMC2772655

[B73] Willsey A. J. Sanders S. J. Li M. Dong S. Tebbenkamp A. T. Muhle R. A. (2013). Coexpression networks implicate human midfetal deep cortical projection neurons in the pathogenesis of autism. *Cell* 155 997–1007. 10.1016/j.cell.2013.10.020 24267886 PMC3995413

[B74] Willsey A. J. State M. W. (2015). Autism spectrum disorders: from genes to neurobiology. *Curr. Opin. Neurobiol.* 30 92–99. 10.1016/j.conb.2014.10.015 25464374 PMC4586254

[B75] Xu X. Wells A. B. O’Brien D. R. Nehorai A. Dougherty J. D. (2014). Cell type-specific expression analysis to identify putative cellular mechanisms for neurogenetic disorders. *J. Neurosci.* 34 1420–1431. 10.1523/JNEUROSCI.4488-13.2014 24453331 PMC3898298

[B76] Young M. D. Wakefield M. J. Smyth G. K. Oshlack A. (2010). Gene ontology analysis for RNA-seq: accounting for selection bias. *Genome Biol.* 11:R14 10.1186/gb-2010-11-2-r14 PMC2872874 20132535

[B77] Yu T. W. Chahrour M. H. Coulter M. E. Jiralerspong S. Okamura-Ikeda K. Ataman B. (2013). Using whole-exome sequencing to identify inherited causes of autism. *Neuron* 77 259–273. 10.1016/j.neuron.2012.11.002 23352163 PMC3694430

[B78] Zeisel A. Muñoz-Manchado A. B. Codeluppi S. Lönnerberg P. Manno G. L. Juréus A. (2015). Cell types in the mouse cortex and hippocampus revealed by single-cell RNA-seq. *Science* 347 1138–1142. 10.1126/science.aaa1934 25700174

[B79] Zeng H. Shen E. H. Hohmann J. G. Oh S. W. Bernard A. Royall J. J. (2012). Large-scale cellular-resolution gene profiling in human neocortex reveals species-specific molecular signatures. *Cell* 149 483–496. 10.1016/j.cell.2012.02.052 22500809 PMC3328777

[B80] Zhang Y. Chen K. Sloan S. A. Bennett M. L. Scholze A. R. O’Keeffe S. (2014). An RNA-sequencing transcriptome and splicing database of glia, neurons, and vascular cells of the cerebral cortex. *J. Neurosci.* 34 11929–11947. 10.1523/JNEUROSCI.1860-14.2014 25186741 PMC4152602

